# Oncologic Outcomes of Surgery in T3 Prostate Cancer: Experience of a Single Tertiary Center

**DOI:** 10.1155/2012/164263

**Published:** 2011-12-19

**Authors:** D. Milonas, G. Smailyte, M. Jievaltas

**Affiliations:** ^1^Department of Urology, Lithuanian University of Health Sciences, 44307 Kaunas, Lithuania; ^2^Institute of Oncology, Vilnius University, 01122 Vilnius, Lithuania

## Abstract

*Aim*. The aim of this study is to present the oncologic outcomes and to determine the prognostic factors of overall survival (OS), cancer-specific survival (CSS), disease-progression-free survival (DPFS), and biochemical-progression-free survival (BPFS) after surgery for pT3 prostate cancer (PCa). *Methods*. Between 2002 and 2007, a pT3 stage after radical prostatectomy was detected in 182 patients at our institution. The Kaplan-Meier analysis was used to calculate OS, CSS, DPFS, and BPFS. Cox regression was used to identify predictive factors of survival. *Results*. pT3a was detected in 126 (69%) and pT3b in 56 (31%) of cases. Five-year OS, CSS, DPFS, and BPFS rates were 90.7%, 94%, 91.8%, and 48.4%, respectively. Survival was significantly different when comparing pT3a to pT3b groups. The 5-year OS, CSS, DPFS, and BPFS were 96% versus 72%, 98% versus 77%, 97.3% versus 79.3%, and 60% versus 24.2%, respectively. Specimen Gleason score was the most significant predictor of OS, CSS, DPFS, and BPFS. The risk of death increased up to 3-fold when a Gleason score 8–10 was present at the final pathology. *Conclusions*. Radical prostatectomy may offer very good CSS, OS, DPFS, and BPFS rates in pT3a PCa. However, outcomes in patients with pT3b or specimen Gleason ≥8 were significantly worse, suggesting the need for multimodality treatment in those cases.

## 1. Introduction

During the last decade, the definition of the optimal treatment in high-risk prostate cancer (PCa) has been among the topics that are of most interest to the urological community, but consensus in this field is still not reached. Up until a decade ago, most T3 PCa patients underwent radiotherapy (RT) or androgen deprivation therapy (ADT) or a combination of both, while only about 36% were initially treated by surgery [1]. Recent publications have revealed that in selected cases of locally advanced and high-grade tumours, surgery as monotherapy or as part of a multimodality treatment may be used instead of RT [2]. The high-risk PCa population, usually described as having prostate specific antigen (PSA) >20 ng/mL, biopsy Gleason score ≥8, or an advanced clinical stage (T3a-b) [3], is however not homogeneous. Recent studies have shown that treatment outcomes can vary widely, depending on whether patients present with only one or rather a combination of those high-risk factors, with the latter patients having the worst outcomes [4–7]. It is still unclear which patients, according to the accepted predictors of aggressive disease behaviour, are the best candidates for surgery, mostly due to the lack of data on long-term oncologic outcomes and randomized clinical trials. According to the European Association of Urology guidelines, surgery is optional in patients presenting with cT3a, Gleason score 8–10, or PSA >20 ng/mL and life expectancy of more than 10 years [8]. Even in highly selected patients with cT3b or cN1 PCa, surgery may be offered as part of a multimodality approach [8]. We believe that radical prostatectomy is indeed an appropriate treatment for more aggressive PCa, but data for confirming that are still insufficient.

The purpose of this study is to present the oncologic outcomes of patients having pT3a and pT3b PCa after surgery, including overall survival (OS), cancer-specific survival (CSS), disease-progression-free survival (DPFS), and biochemical-progression-free survival (BPFS). Furthermore, we aimed to analyze predictive parameters in survival.

## 2. Material and Methods

During the period 2002–2007, 840 radical retropubic prostatectomies (RRP) were performed in our tertiary referral institution. 192 of them had pathological stage T3 (22.9%). Ten patients were lost for additional followup. Final analysis was carried out using the data of 182 patients with complete followup. No patients received neoadjuvant treatment. The last PSA before biopsy was used for analysis.

Biopsy Gleason score ≥7, PSA >10 ng/mL, or clinical stage T3 was indication for lymph node removal. 113 of 182 (62.1%) patients of our study population had such criteria. For the other 69 (37.9%) patients, a lymphadenectomy was not performed.

The pathological examination of radical prostatectomy specimens and bilateral pelvic lymph nodes was performed by one dedicated uropathologist.

Serum PSA and physical examination were performed every 3 months in the first year after surgery, every 6 months in the second and third years, and annually thereafter. The PSA data were taken from outpatient clinic files. Data about patients' death and cause of death were received from the National Cancer Registry.

OS was defined as the time from surgery to death from any cause. CSS was defined as the time from surgery to death caused by PCa or complications of this disease. Biochemical progression was defined as the time from surgery to PSA level ≥0.2 ng/mL confirmed by repeated test. Disease progression was defined as the development of either local disease recurrence or distant metastasis. Adjuvant treatment was defined as either ADT or RT given within 3 months after surgery. Salvage treatment was defined as any kind of therapy (RT or ADT) given later than 3 months after surgery. The main indication for adjuvant treatment was positive lymph nodes. Combination of Gleason score ≥8, preoperative PSA >20 ng/mL, pT3b, and positive surgical margins were other indicators for adjuvant treatment.

The Kaplan-Meier survival analysis was used to calculate the OS, CSS, DPFS, and BPFS. The differences were tested by log-rank test. The Cox regression analysis was used to determine the prognostic factors for survival.

## 3. Results

An overview of the patients' preoperative and postoperative parameters is shown in Table 1. The median followup was 54 months (range 6–96). 5-year rates for OS, CSS, DPFS, and BPFS in our study cohort were 90.7%, 94%, 91.8%, and, 48.4%, respectively. Cox regression analysis revealed that from all parameters (age, biopsy and surgery Gleason score, surgical margin and lymph node status, pathological stage, and preoperative PSA level) only pathological stage and postoperative Gleason score had an impact on overall mortality and disease progression (Table 2). The Gleason score also has the strongest impact on CSS. According to Cox regression analysis, there were no other parameters influencing cancer specific mortality (Table 2). Pathological stage, lymph node status and postoperative Gleason score were the strongest prognostic factors for biochemical disease progression (Table 2).

### 3.1. Lymph Node Status

**Figure 1:**
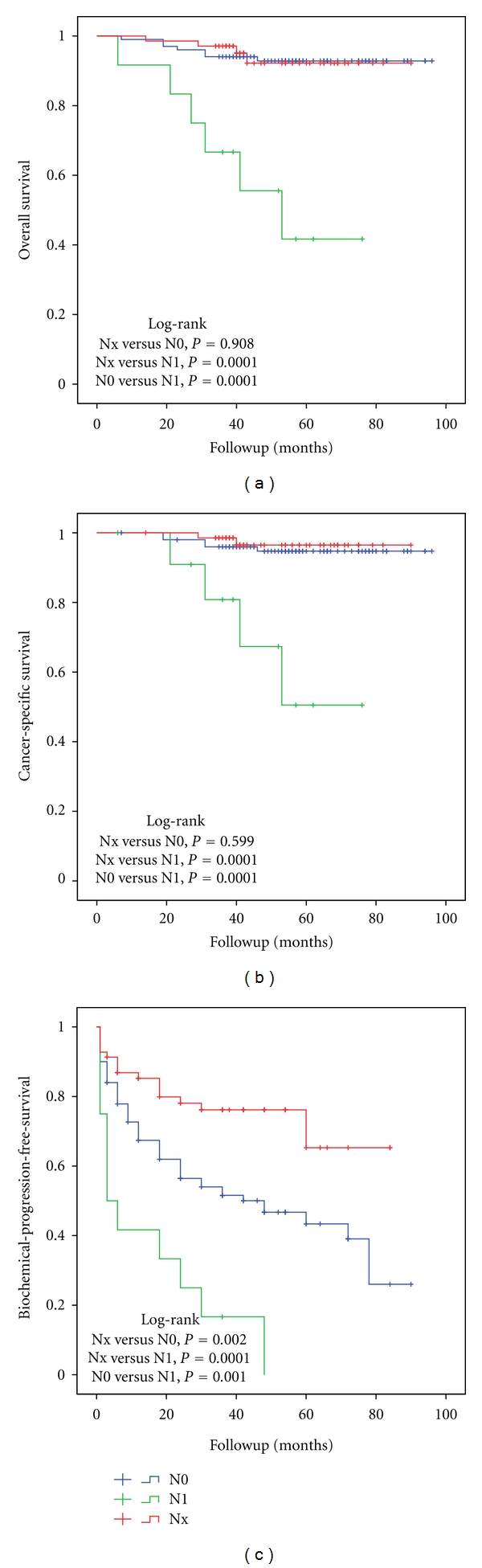
The Kaplan-Meier analysis with log-rank test for overall (a), cancer-specific survival (b), and biochemical-progression-free survival (c) stratified for lymph node status.

A mean of 6.4 (range 1–15) lymph nodes were removed, and the overall positive node detection rate was 10.6%. During the study period, the overall mortality rate in pN1 patients was 50% and cancer-specific mortality rate was 33.3%. Patients with pN0 or pNx had significantly lower overall (6.9% and 5.8%, resp.) and cancer-specific mortality rate (5.0% and 2.9%, resp.). The Kaplan-Meier analysis showed that 5-year OS (93% versus 40%, Figure 1(a)), CSS (95% versus 50%, Figure 1(b)), and DPFS (96.5% versus 92.6% versus 40.7%) rates were significantly different when comparing pNx, pN0, and pN1, respectively. There was no difference between pNx and pN0 in survival analysis. PSA relapse rate was different comparing patients with pN1, pN0, and pNx. 5-year BPFS was 0% in pN1 group, 43.4% in pN0, and 65.3% in pNx groups (Figure 1(c)).

### 3.2. Pathological Stage

**Figure 2:**
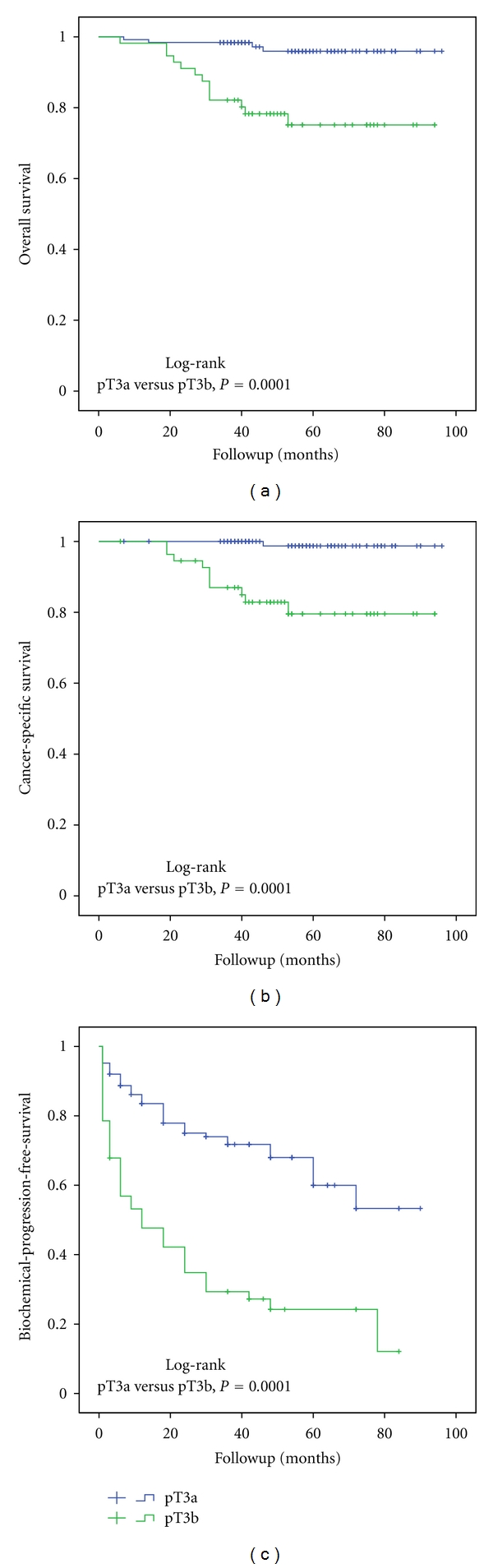
The Kaplan-Meier analysis with log-rank test for overall (a), cancer-specific survival (b), and biochemical-progression-free survival (c) stratified for pathological stage.

The Kaplan-Meier analysis showed that pT3a and pT3b stages provide significantly different 5-year OS (96% versus 72%, resp.; Figure 2(a)), CSS (98% versus 77%, resp.; Figure 2(b)), DPFS (97.3% versus 79.3%, resp.) and BPFS (60% versus 24.2%, resp.; Figure 2(c)). Positive lymph nodes were found significantly less frequently in pT3a (2 of 71, 2.8%) than pT3b PCa (10 of 42, 23.8%) (*P* = 0.0001). Lymph node positivity did not impact survival in the stage pT3a PCa, but had a significant role in the stage pT3b PCa. Estimated 5-year OS, CSS, and DPFS rates in pT3bN1 (38%, 50%, and 38.6%, resp.) were significantly worse (*P* = 0.0001) compared with pT3bN0-Nx (84%, 88%, and 86.2%, resp.). 5-year BPFS rate was 31% in patients with pT3bN0-Nx while all patients with pT3bN1 had biochemical relapse during the study period.

### 3.3. Gleason Score

**Figure 3:**
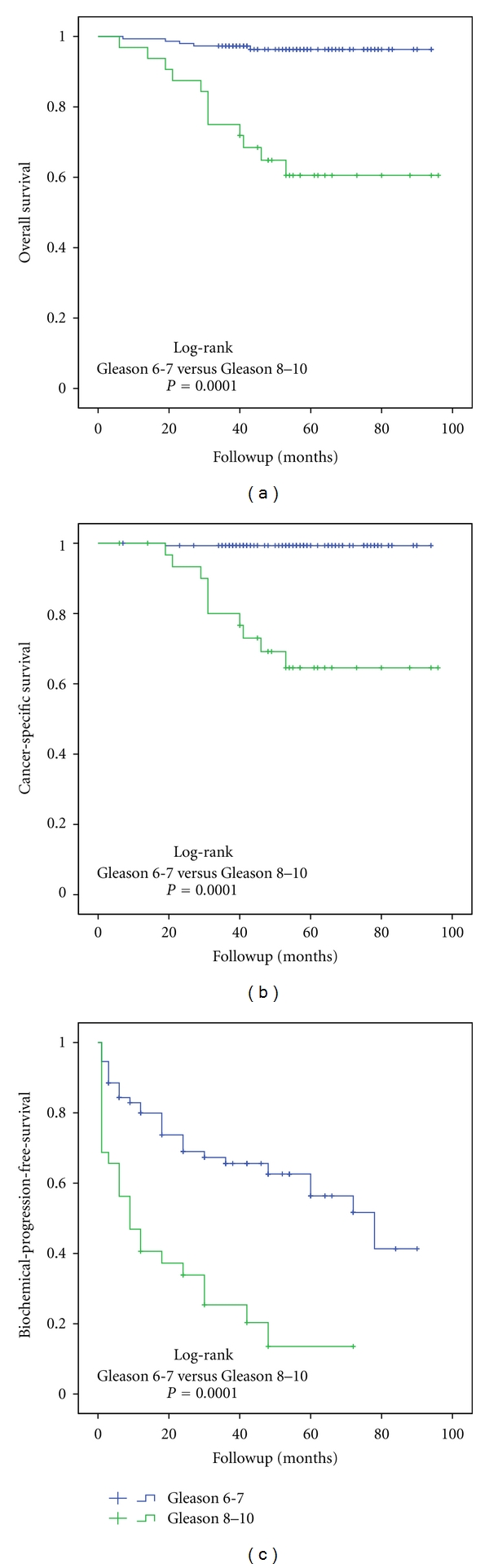
The Kaplan-Meier analysis with log-rank test for overall (a), cancer-specific survival (b), and biochemical-progression-free survival (c) stratified for Gleason score sum.

During the study, close correlation between pathological stage and cancer differentiation was established. The mean biopsy Gleason score was significantly worse in pT3b compared to pT3a PCa (6.8 versus 6.4, *P* = 0.001) and after surgery (7.5 versus 6.9, *P* = 0.001). Gleason score upgrading was detected in 52.5% of cases and downgrading in 5.6% of cases. Increased Gleason score was correlated with an increased positive lymph node rate: 29.2% at Gleason ≥8 versus 5.7% at Gleason ≤7 (*P* = 0.003). The Kaplan-Meier analysis demonstrates significant differences between Gleason ≤7 and ≥8 for OS (Figure 3(a)), CSS (Figure 3(b)), DPFS, and BPFS (Figure 3(c)) in the total study population. The estimated 5-year OS, CSS, DPFS, and BPFS rates in patients with Gleason score ≥8 were 61%, 64%, 62.4%, and 13.5%, respectively, while in Gleason score ≤7, 5-year OS, CSS, DPFS, and BPFS were 96%, 99%, 97.8%, and 56.3%, respectively.

### 3.4. Surgical Margin Status

Positive surgical margin (R1) rate was significantly different (*P* = 0.03) comparing pT3a to pT3b cases (Table 1). Although in Cox regression this parameter was not determined as prognostic factor for survival the Kaplan-Meier analysis demonstrated different 5-year CSS (98.6% versus 90%, log-rank *P* = 0.047) and BPFS (55.9% versus 44.2%, log-rank *P* = 0.08) rates comparing R0 to R1 in all study population. Impact of surgical margin status on outcome was analyzed separately in patients who did not receive adjuvant treatment. Only 5-year BPFS rate was different comparing patients with R0 to those with R1: 62.3% versus 52.5% (log-rank *P* = 0.023), respectively.

### 3.5. Postoperative Treatment

Patients with pT3 PCa are generally considered at risk for disease progression. Therefore, adjuvant or salvage treatment (RT or ADT) is often applied. In our study population, additional treatment was given to 32.4% (adjuvant to 15.9% and salvage to 16.5%) of cases: 21.4% in the pT3a and 57.1% in the pT3b subgroups. 20.3% of patients received ADT, 7.1% RT, and 5% RT with ADT. All twelve patients with N1 received adjuvant treatment: two of them received RT with ADT and the other ten ADT alone.

## 4. Discussion

During the last decade, the discussion about the role of surgery in locally advanced PCa became increasingly active. Before that time, treatment of locally advanced PCa was mostly in hands of radiation oncologists [1]. Such discussion became possible for several reasons: successful treatment of high-risk PCa with RT monotherapy requires high radiation doses (74–80 Gy), leading to higher rates of adverse events. On the other hand, recent studies [2, 9–12] demonstrate outcomes after surgery which can be compared with radiation therapy +/− ADT. Our single center study shows that surgical treatment may indeed be a reasonable treatment option in locally advanced PCa with 90.7% OS and 94% CSS at the 5-year follow-up mark. Surgery in pT3a PCa, independently of cancer differentiation and PSA level demonstrated significantly higher 5-year OS, CSS, DPFS, and BPFS rates when compared to pT3b disease (96% versus 72%, 98% versus 77%, 97.3% versus 79.3%, and 60% versus 24.2%, resp.). The survival rates of the pT3a patients in our study are similar to those reported by Hsu et al. in a study of 200 patients with unilateral cT3a treated by surgery. They also showed that progression-free survival rates of patients with pT3a PCa did not differ significantly from those with pT2 disease [7]. Some other authors have also reported their outcomes of surgical treatment for T3 PCa. Summarizing those results, 5-year CSS and OS rates varied from 85 to 100% and from 75 to 98%, respectively [9–12]. Direct comparison between the outcomes of surgery and radiation is inadequate because of inherent selection biases, Gleason score upgrading, or stage migration after surgery. Nevertheless, this issue could be partially solved using data from the RTOG trials which compared RT to a combined approach using RT and ADT [13]. In a review of those RTOG trials, different PCa risk groups were identified with group 2 (Gleason ≤6, T3Nx-N1 or Gleason 7, T1-2Nx) and group 3 (Gleason 7, T3Nx-N1 or Gleason ≥8, T1-2Nx) most closely corresponding with our study population. After radiation, the 5-year OS and CSS rates were 82% and 94% for group 2 and 68% and 83% for group 3, respectively [13]. Outcomes from another long-term study comparing RT to RT with concomitant ADT were reported by Bolla et al. [14]. In the EORTC trial, 412 patients with locally advanced PCa were treated with RT alone or in combination with ADT. Five-year OS and CSS rates were, respectively, 62 and 79% in the radiation-alone group. Better survival was reported in combination group: 78% and 94%, respectively. Our study data showed a comparable 94% 5-year CSS, similar to RT and ADT combination therapy.

The group of locally advanced PCa is heterogeneous. PSA and specimen Gleason score have a significant impact on the survival analysis. According to our study, pT3a patients with a PSA <10 ng/mL had significantly better OS and BPFS when compared to those with a PSA level >20 ng/mL (log rank *P* = 0.048 and *P* = 0.0001, resp.). Patients with a PSA level of 10 to 20 ng/mL did not have significantly different OS when compared to PSA <10 or PSA >20 ng/mL (log rank *P* = 0.552) but had different BPFS compared to PSA >20 ng/mL (log rank *P* = 0.008). In the pT3a group, PSA had no impact on CSS and DPFS. In the pT3b group, we found no significant impact of PSA level on the 5-year OS, CSS, or DPFS. A possible explanation for this observation could be the variable application of adjuvant therapies. 5-year BPFS rate in the pT3b group was different comparing patients with PSA >20 to <10 ng/mL (log rank *P* = 0.019). Some recent studies also studied the role of PSA in survival and biochemical or disease progression [4, 5, 15]. All authors agreed that PSA >20 ng/mL indeed could be considered as a high-risk factor. Our findings support that patients with PSA >20 ng/mL had significantly worse BPFS and OS but not CSS or DPFS rates in pT3 PCa population.

Gleason score has long been recognized as an important risk indicator for worse outcome. In locally advanced PCa, biopsy Gleason sum has a tendency to be upgraded, and in our series upgrading was indeed frequent (up to 50%). In fact, in our study, specimen Gleason score was identified as the most important outcome predictor. Our data showed a significant difference between survival curves comparing Gleason score 5–7 to 8–10. More importantly, patients with postoperative Gleason ≥8 are associated with a 2.8-fold increased risk of death and 2.4-fold increased risk of disease progression. If cancer differentiation after surgery is ≥8, the risk of death from cancer increases more than 3-fold. Gleason score 8–10 is also associated with a higher node-positive rate when compared with Gleason score 7 (6.3% versus 4.5%, chi-square test *P* = 0.03). Most of the published studies confirm that Gleason score 8–10 indeed determines worse biochemical or disease-free survival [6, 16, 17] both in locally advanced and organ-confined disease [18]. Our study shows that 5-year OS, CSS, DPFS, and BPFS rates in Gleason score 8–10 PCa were 61%, 64%, 62.4%, and 13.5% compared to 96%, 99%, 97.8%, and 56.3% if Gleason score was 5–7. However, significant survival differences between high- and moderate-grade PCa do not mean that a more advanced tumor grade is a contraindication for surgery. Tewari et al. pointed out that long-term results in high-grade PCa after surgery are better when comparing surgically treated patients with those who underwent RT or conservative treatment [19]. In 453 patients with biopsy Gleason 8–10, median OS after surgery was 9.7 years, while for radiation this was 6.7 years and for conservative treatment 5.2 years. The risk of cancer-related death after surgery was 68% lower than after conservative treatment and 48% lower than after RT.

The pT3b stage is associated with the poorest oncological outcomes after surgery. In our study, the rate of positive margins was 71.7%, while 23.8% and 38.2% had pN1 disease and specimen Gleason score 8–10, respectively. These adverse pathological outcomes are directly related to the oncological outcomes: 5-year CSS was 77%, OS was 72%, DPFS was 79.3%, and BPFS was 24.2%. A subanalysis of T3b patients without positive lymph nodes (pT3bN0-Nx) showed 5-year OS, CSS, DPFS, and BPFS rates of 84%, 88%, 94%, and 52.1%, respectively. There are no possibilities to compare the results of surgery and RT in such small cohort of patients. If we look to the outcomes (5-year OS rates >75% and CSS >85%) of radical prostatectomy at advanced stage and high-grade PCa in large review presented by Van Poppel and Joniau [2], our pT3b survival data are similar. This suggests that not all patients with cancer extending into the seminal vesicles are destined to have poor outcomes. Lymph node status and Gleason score seem to play the most important role in pT3b PCa outcomes.

From our analysis the presence of positive surgical margins was not significant predictor for survival in Cox regression. The Kaplan-Meier analysis showed that only 5-year BPFS was different comparing R0 to R1 in all study cohort (55.9% versus 44.2%, log rank *P* = 0.08). We found similar data excluding patients who received adjuvant treatment. Only BPFS was different comparing surgical margin status. The similar findings were reported by Hsu et al. [7]. Authors concluded that margin status was a significant independent predictor in BPFS but did not influence OS, CSS, and DPFS. The question remains if patients with positive margins should receive adjuvant treatment in pT3 cases. Our study data confirms that R1 with Gleason ≥8 is proper candidate for adjuvant treatment, but more randomized studies are needed to cover this topic.

Generally, it is accepted that patients with locally advanced PCa at final histology are ideal candidates for additional treatment after surgery. Up until now, there is still no consensus on which treatment modality—RT, ADT, or a combination—is the best choice to decrease the risk of disease progression following surgery. In the present study, only 32.4% of cases (21.4% in pT3a and 57.1% in pT3b) received additional treatment: 15.9 received adjuvant and 16.5% salvage treatment. Cox regression analysis did not show impact of adjuvant therapy on survival, but we were unable to investigate real influence of adjuvant treatment on outcomes because of small number of cases and not randomized study design. According to our data, 42.9% of patients in pT3b and 78.6% in pT3a did not receive any additional treatment during median 4.5-year followup. It shows that surgery as monotherapy could be discussed with patient even in suspected T3 PCa.

With 5-year OS, CSS, DPFS, and BPFS of 91%, 94%, 91.8%, and 48.4%, our study supports the notion that radical prostatectomy with adjuvant or salvage therapy as RT plus ADT when needed may provide comparable outcomes in locally advanced PCa, especially in pT3a. However, this finding should be confirmed in prospective, randomized studies.

## 5. Conclusions

Radical prostatectomy may offer very good CSS, OS, DPFS, and BPFS rates in pT3a PCa. However, outcomes in patients with pT3b or specimen Gleason ≥8 were significantly worse, suggesting the need for multimodality treatment in those cases.

## Figures and Tables

**Table 1:** Patient characteristics.

Parameter	pT3a (*N* = 126, 69.2%)	pT3b (*N* = 56, 30.8%)	All (*N* = 182, 100%)
Median age (yr) (range)	66.5 (49–78)	65 (48–76)	66 (48–78)
Median PSA (ng/mL) (range)	7.63 (0.68–39.89)	11.6 (3.1–98.4)	8.67 (0.68–98.4)
Mean biopsy Gleason (range) Gleason ≤6 Gleason 7 Gleason ≥8	6.4 (6–9) 68.3% 26.0% 5.7%	6.8 (5–10) 41.1% 41.1% 17.9%	6.5 (5–10) 59.8% 30.7% 9.5%
Mean surgery Gleason (range) Gleason ≤6 Gleason 7 Gleason ≥8	6.9 (6–9) 19.8% 71.4% 8.8%	7.5 (6–9) 3.6% 58.2% 38.2%	7.1 (6–9) 14.9% 67.4% 17.7%
*R* (%)	54.2%	71.7%	59.5%
*N*+ (rate)	2.8% (2/71)	23.8% (10/42)	10.6% (12/113)
PSA relapse	29.6%	75.0%	43.6%
Deaths (rate)	3.2% (4/126)	23.2% (13/56)	9.3% (17/182)
Deaths from cancer (rate)	0.8% (1/126)	17.9% (10/56)	6% (11/182)
mts	2.4%	17.9%	7.1%
Median followup (mo) (range)	56 (7–96)	50.5 (6–94)	54 (6–96)

**Table 2:** Cox multivariate regression analysis of preoperative and histopathologic parameters.

Parameter	Overall survival			Cancer-specific survival			Biochemical progression free survival		
	HR	95% CI	*P* value	HR	95% CI	*P* value	HR	95% CI	*P* value
Pathological stage	0.195	0.052–0.735	0.016	0.00	0.00–9.80	0.923	0.475	0.291–0.775	0.003
Age	1.06	0.977–1.152	0.162	1.068	0.943–1.208	0.29	1.005	0.969–1.043	0.779
Lymph node	0.546	0.158–1.88	0.337	0.832	0.166–4.276	0.823	0.715	0.542–0.943	0.018
Pre operative PSA	1.013	0.982–1.046	0.406	1.007	0.964–1.051	0.766	1.005	0.991–1.019	0.490
Surgical margins	0.878	0.220–3.514	0.855	0.522	0.058–4.686	0.562	0.756	0.440–1.30	0.312
Biopsy Gl. score	1.072	0.600–1.915	0.814	1.093	0.530–2.251	0.81	1.077	0.792–1.466	0.636
Surgery Gl. score	2.82	1.492–5.337	0.001	3.24	1.018–10.311	0.04	2.029	1.461–2.818	0.0001
